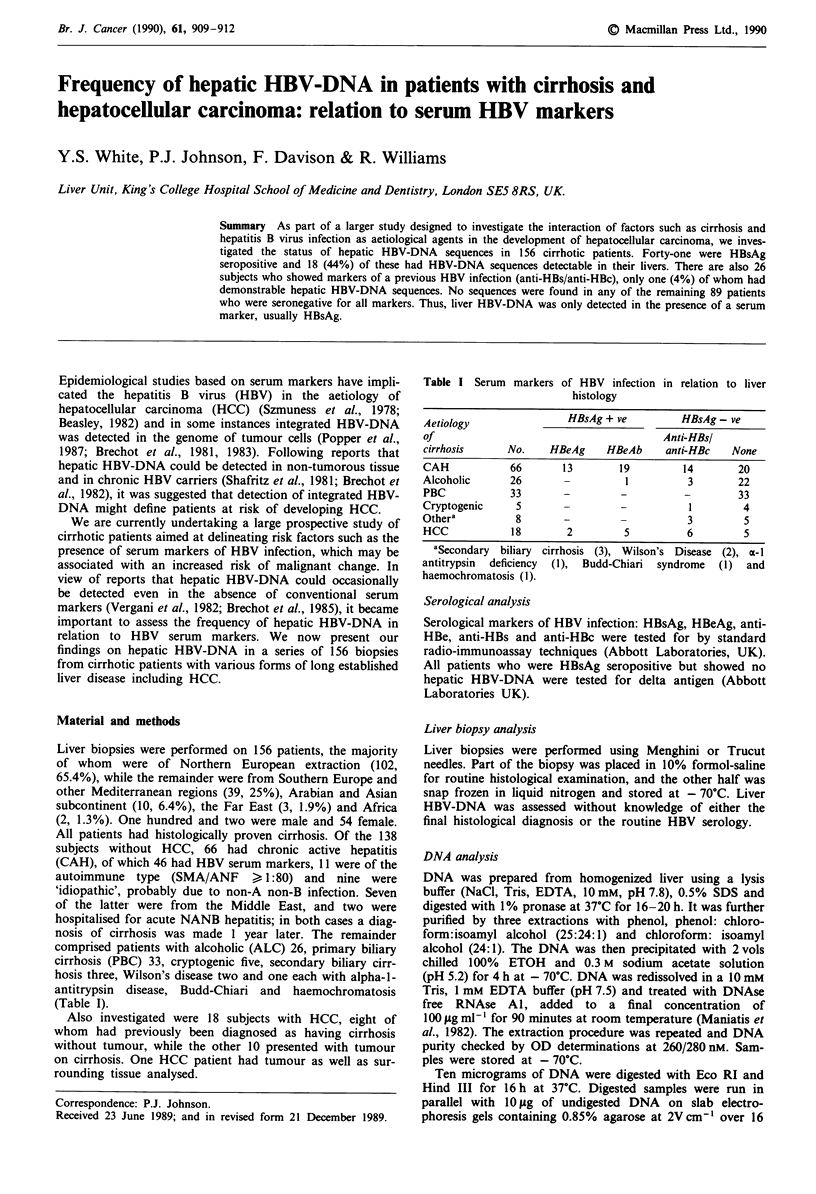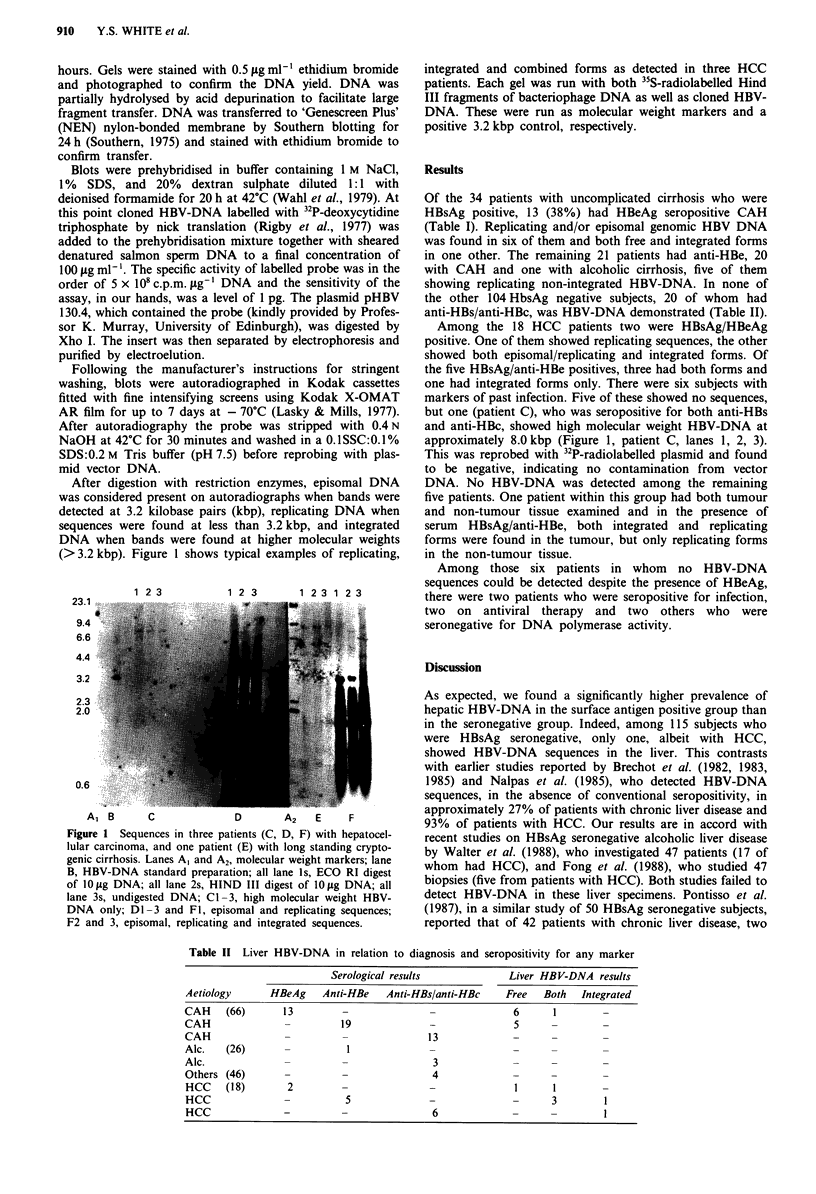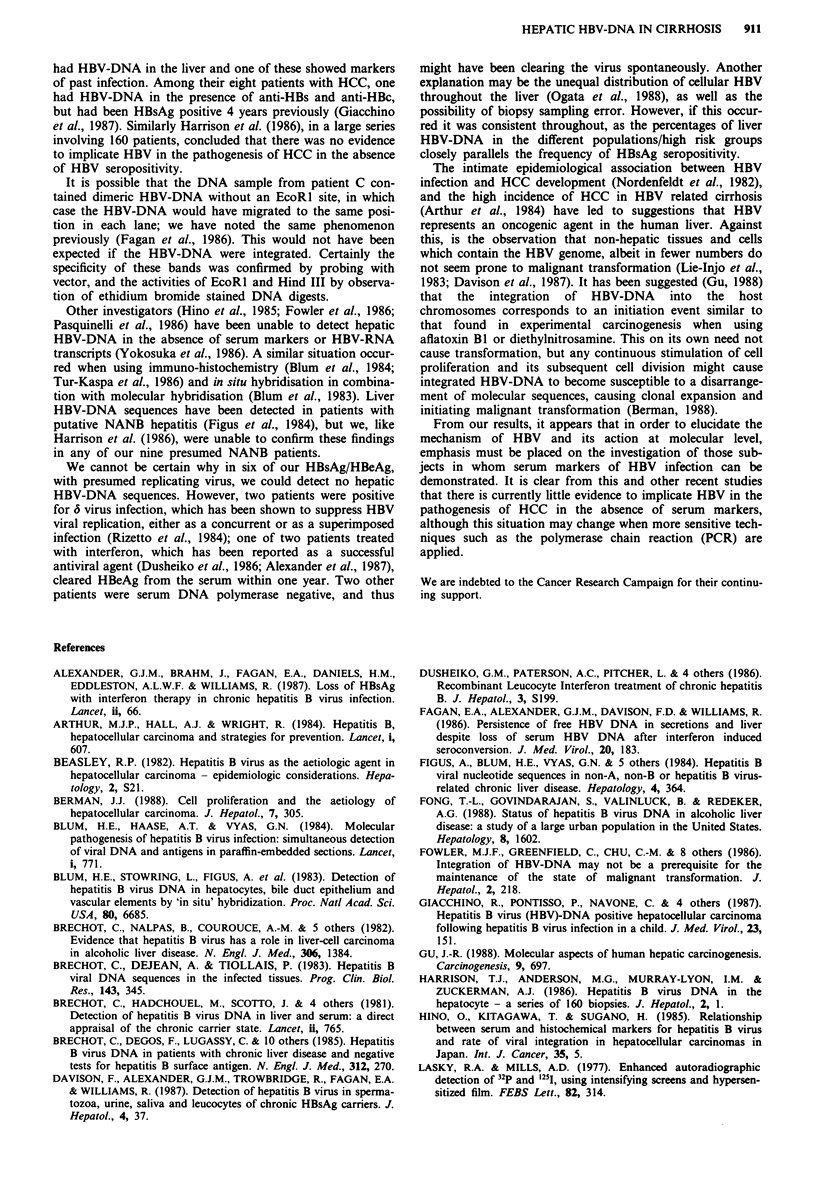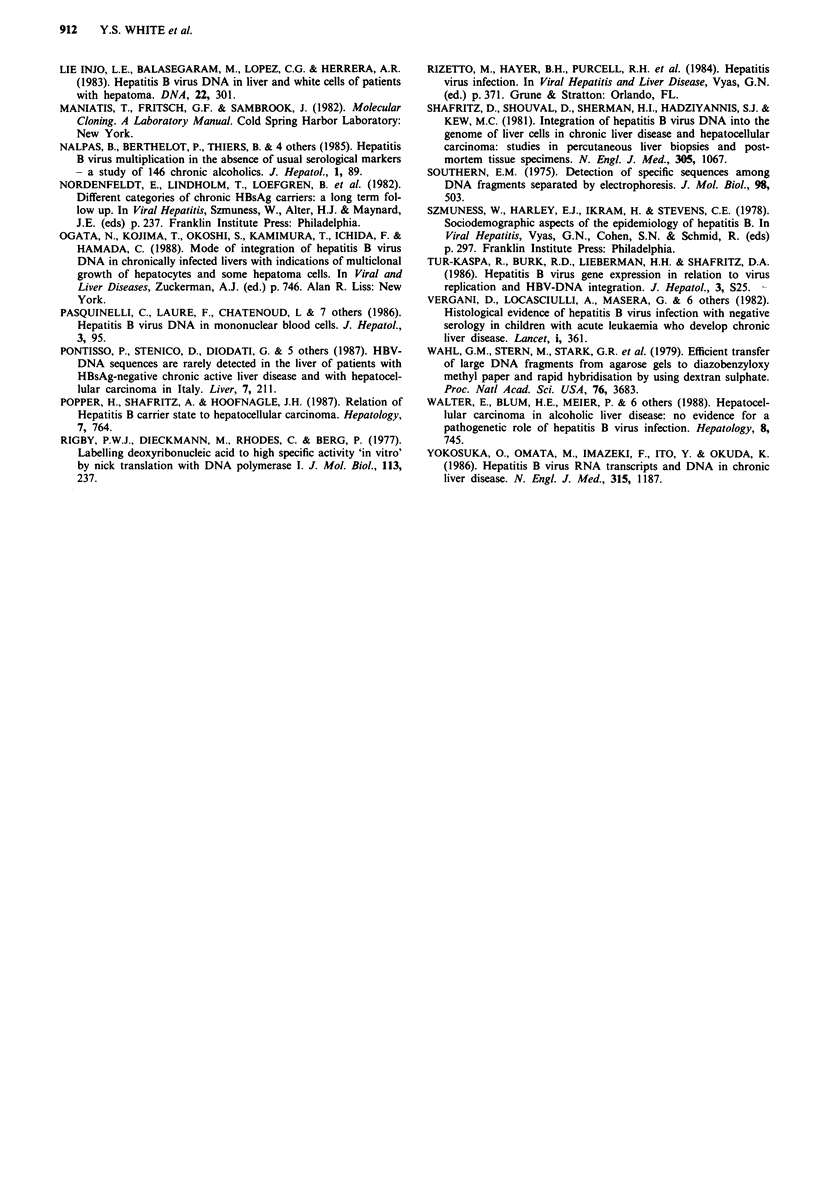# Frequency of hepatic HBV-DNA in patients with cirrhosis and hepatocellular carcinoma: relation to serum HBV markers.

**DOI:** 10.1038/bjc.1990.203

**Published:** 1990-06

**Authors:** Y. S. White, P. J. Johnson, F. Davison, R. Williams

**Affiliations:** Liver Unit, King's College Hospital School of Medicine and Dentistry, London, UK.

## Abstract

**Images:**


 © Macmillan Press Ltd., 1990

### Frequency of hepatic HBV-DNA in patients with cirrhosis and hepatocellular carcinoma: relation to serum HBV markers

Y.S. White, P.J. Johnson, F. Davison & R. Williams

*Liver Unit, King's College Hospital School of Medicine and Dentistry, London SE5 8RS, UK.*

**Summary** As part of a larger study designed to investigate the interaction of factors such as cirrhosis and hepatitis B virus infection as aetiological agents in the development of hepatocellular carcinoma, we investigated the status of hepatic HBV-DNA sequences in 156 cirrhotic patients. Forty-one were HBsAg seropositive and 18 (44%) of these had HBV-DNA sequences detectable in their livers. There are also 26 subjects who showed markers of a previous HBV infection (anti-HBs/anti-HBc), only one (4%) of whom had demonstrable hepatic HBV-DNA sequences. No sequences were found in any of the remaining 89 patients who were seronegative for all markers. Thus, liver HBV-DNA was only detected in the presence of a serum marker, usually HBsAg.

Epidemiological studies based on serum markers have implicated the hepatitis B virus (HBV) in the aetiology of hepatocellular carcinoma (HCC) (Szmuness *et al.*, 1978; Beasley, 1982) and in some instances integrated HBV-DNA was detected in the genome of tumour cells (Popper *et al.*, 1987; Brechot *et al.*, 1981, 1983). Following reports that hepatic HBV-DNA could be detected in non-tumorous tissue and in chronic HBV carriers (Shafritz *et al.*, 1981; Brechot *et al.*, 1982), it was suggested that detection of integrated HBV-DNA might define patients at risk of developing HCC.

We are currently undertaking a large prospective study of cirrhotic patients aimed at delineating risk factors such as the presence of serum markers of HBV infection, which may be associated with an increased risk of malignant change. In view of reports that hepatic HBV-DNA could occasionally be detected even in the absence of conventional serum markers (Vergani *et al.*, 1982; Brechot *et al.*, 1985), it became important to assess the frequency of hepatic HBV-DNA in relation to HBV serum markers. We now present our findings on hepatic HBV-DNA in a series of 156 biopsies from cirrhotic patients with various forms of long established liver disease including HCC.

### Material and methods

Liver biopsies were performed on 156 patients, the majority of whom were of Northern European extraction (102, 65.4%), while the remainder were from Southern Europe and other Mediterranean regions (39, 25%), Arabian and Asian subcontinent (10, 6.4%), the Far East (3, 1.9%) and Africa (2, 1.3%). One hundred and two were male and 54 female. All patients had histologically proven cirrhosis. Of the 138 subjects without HCC, 66 had chronic active hepatitis (CAH), of which 46 had HBV serum markers, 11 were of the autoimmune type (SMA/ANF $\geqslant$1:80) and nine were 'idiopathic', probably due to non-A non-B infection. Seven of the latter were from the Middle East, and two were hospitalised for acute NANB hepatitis; in both cases a diagnosis of cirrhosis was made 1 year later. The remainder comprised patients with alcoholic (ALC) 26, primary biliary cirrhosis (PBC) 33, cryptogenic five, secondary biliary cirrhosis three, Wilson's disease two and one each with alpha-1-antitrypsin disease, Budd-Chiari and haemochromatosis (Table I).

Also investigated were 18 subjects with HCC, eight of whom had previously been diagnosed as having cirrhosis without tumour, while the other 10 presented with tumour on cirrhosis. One HCC patient had tumour as well as surrounding tissue analysed.

Correspondence: P.J. Johnson.
Received 23 June 1989; and in revised form 21 December 1989.

**Table I** Serum markers of HBV infection in relation to liver histology

| *Aetiology of cirrhosis* | *No.* | *HBsAg + ve* | | *HBsAg − ve* | |
|---|---|---|---|---|---|
| | | *HBeAg* | *HBeAb* | *Anti-HBs/ anti-HBc* | *None* |
| CAH | 66 | 13 | 19 | 14 | 20 |
| Alcoholic | 26 | – | 1 | 3 | 22 |
| PBC | 33 | – | – | – | 33 |
| Cryptogenic | 5 | – | – | 1 | 4 |
| Other[a] | 8 | – | – | 3 | 5 |
| HCC | 18 | 2 | 5 | 6 | 5 |

[a]Secondary biliary cirrhosis (3), Wilson's Disease (2), α-1 antitrypsin deficiency (1), Budd-Chiari syndrome (1) and haemochromatosis (1).

### *Serological analysis*

Serological markers of HBV infection: HBsAg, HBeAg, anti-HBe, anti-HBs and anti-HBc were tested for by standard radio-immunoassay techniques (Abbott Laboratories, UK). All patients who were HBsAg seropositive but showed no hepatic HBV-DNA were tested for delta antigen (Abbott Laboratories UK).

### *Liver biopsy analysis*

Liver biopsies were performed using Menghini or Trucut needles. Part of the biopsy was placed in 10% formol-saline for routine histological examination, and the other half was snap frozen in liquid nitrogen and stored at − 70°C. Liver HBV-DNA was assessed without knowledge of either the final histological diagnosis or the routine HBV serology.

### *DNA analysis*

DNA was prepared from homogenized liver using a lysis buffer (NaCl, Tris, EDTA, 10 mM, pH 7.8), 0.5% SDS and digested with 1% pronase at 37°C for 16–20 h. It was further purified by three extractions with phenol, phenol: chloroform:isoamyl alcohol (25:24:1) and chloroform: isoamyl alcohol (24:1). The DNA was then precipitated with 2 vols chilled 100% ETOH and 0.3 M sodium acetate solution (pH 5.2) for 4 h at − 70°C. DNA was redissolved in a 10 mM Tris, 1 mM EDTA buffer (pH 7.5) and treated with DNAse free RNAse A1, added to a final concentration of 100 $\mu g\ ml^{-1}$ for 90 minutes at room temperature (Maniatis *et al.*, 1982). The extraction procedure was repeated and DNA purity checked by OD determinations at 260/280 nM. Samples were stored at − 70°C.

Ten micrograms of DNA were digested with Eco RI and Hind III for 16 h at 37°C. Digested samples were run in parallel with 10 $\mu g$ of undigested DNA on slab electrophoresis gels containing 0.85% agarose at 2V $cm^{-1}$ over 16

hours. Gels were stained with 0.5 μg ml$^{-1}$ ethidium bromide and photographed to confirm the DNA yield. DNA was partially hydrolysed by acid depurination to facilitate large fragment transfer. DNA was transferred to 'Genescreen Plus' (NEN) nylon-bonded membrane by Southern blotting for 24 h (Southern, 1975) and stained with ethidium bromide to confirm transfer.

Blots were prehybridised in buffer containing 1 M NaCl, 1% SDS, and 20% dextran sulphate diluted 1:1 with deionised formamide for 20 h at 42°C (Wahl *et al.*, 1979). At this point cloned HBV-DNA labelled with $^{32}$P-deoxycytidine triphosphate by nick translation (Rigby *et al.*, 1977) was added to the prehybridisation mixture together with sheared denatured salmon sperm DNA to a final concentration of 100 μg ml$^{-1}$. The specific activity of labelled probe was in the order of $5 \times 10^8$ c.p.m. μg$^{-1}$ DNA and the sensitivity of the assay, in our hands, was a level of 1 pg. The plasmid pHBV 130.4, which contained the probe (kindly provided by Professor K. Murray, University of Edinburgh), was digested by Xho I. The insert was then separated by electrophoresis and purified by electroelution.

Following the manufacturer's instructions for stringent washing, blots were autoradiographed in Kodak cassettes fitted with fine intensifying screens using Kodak X-OMAT AR film for up to 7 days at − 70°C (Lasky & Mills, 1977). After autoradiography the probe was stripped with 0.4 N NaOH at 42°C for 30 minutes and washed in a 0.1SSC:0.1% SDS:0.2 M Tris buffer (pH 7.5) before reprobing with plasmid vector DNA.

After digestion with restriction enzymes, episomal DNA was considered present on autoradiographs when bands were detected at 3.2 kilobase pairs (kbp), replicating DNA when sequences were found at less than 3.2 kbp, and integrated DNA when bands were found at higher molecular weights (>3.2 kbp). Figure 1 shows typical examples of replicating, integrated and combined forms as detected in three HCC patients. Each gel was run with both $^{35}$S-radiolabelled Hind III fragments of bacteriophage DNA as well as cloned HBV-DNA. These were run as molecular weight markers and a positive 3.2 kbp control, respectively.

**Figure 1** Sequences in three patients (C, D, F) with hepatocellular carcinoma, and one patient (E) with long standing cryptogenic cirrhosis. Lanes $A_1$ and $A_2$, molecular weight markers; lane B, HBV-DNA standard preparation; all lane 1s, ECO RI digest of 10 μg DNA; all lane 2s, HIND III digest of 10 μg DNA; all lane 3s, undigested DNA; C1–3, high molecular weight HBV-DNA only; D1–3 and F1, episomal and replicating sequences; F2 and 3, episomal, replicating and integrated sequences.

### Results

Of the 34 patients with uncomplicated cirrhosis who were HBsAg positive, 13 (38%) had HBeAg seropositive CAH (Table I). Replicating and/or episomal genomic HBV DNA was found in six of them and both free and integrated forms in one other. The remaining 21 patients had anti-HBe, 20 with CAH and one with alcoholic cirrhosis, five of them showing replicating non-integrated HBV-DNA. In none of the other 104 HbsAg negative subjects, 20 of whom had anti-HBs/anti-HBc, was HBV-DNA demonstrated (Table II).

Among the 18 HCC patients two were HBsAg/HBeAg positive. One of them showed replicating sequences, the other showed both episomal/replicating and integrated forms. Of the five HBsAg/anti-HBe positives, three had both forms and one had integrated forms only. There were six subjects with markers of past infection. Five of these showed no sequences, but one (patient C), who was seropositive for both anti-HBs and anti-HBc, showed high molecular weight HBV-DNA at approximately 8.0 kbp (Figure 1, patient C, lanes 1, 2, 3). This was reprobed with $^{32}$P-radiolabelled plasmid and found to be negative, indicating no contamination from vector DNA. No HBV-DNA was detected among the remaining five patients. One patient within this group had both tumour and non-tumour tissue examined and in the presence of serum HBsAg/anti-HBe, both integrated and replicating forms were found in the tumour, but only replicating forms in the non-tumour tissue.

Among those six patients in whom no HBV-DNA sequences could be detected despite the presence of HBeAg, there were two patients who were seropositive for infection, two on antiviral therapy and two others who were seronegative for DNA polymerase activity.

### Discussion

As expected, we found a significantly higher prevalence of hepatic HBV-DNA in the surface antigen positive group than in the seronegative group. Indeed, among 115 subjects who were HBsAg seronegative, only one, albeit with HCC, showed HBV-DNA sequences in the liver. This contrasts with earlier studies reported by Brechot *et al.* (1982, 1983, 1985) and Nalpas *et al.* (1985), who detected HBV-DNA sequences, in the absence of conventional seropositivity, in approximately 27% of patients with chronic liver disease and 93% of patients with HCC. Our results are in accord with recent studies on HBsAg seronegative alcoholic liver disease by Walter *et al.* (1988), who investigated 47 patients (17 of whom had HCC), and Fong *et al.* (1988), who studied 47 biopsies (five from patients with HCC). Both studies failed to detect HBV-DNA in these liver specimens. Pontisso *et al.* (1987), in a similar study of 50 HBsAg seronegative subjects, reported that of 42 patients with chronic liver disease, two

**Table II** Liver HBV-DNA in relation to diagnosis and seropositivity for any marker

| | *Serological results* | | | *Liver HBV-DNA results* | | |
|---|---|---|---|---|---|---|
| *Aetiology* | *HBeAg* | *Anti-HBe* | *Anti-HBs/anti-HBc* | *Free* | *Both* | *Integrated* |
| CAH (66) | 13 | – | – | 6 | 1 | – |
| CAH | – | 19 | – | 5 | – | – |
| CAH | – | – | 13 | – | – | – |
| Alc. (26) | – | 1 | – | – | – | – |
| Alc. | – | – | 3 | – | – | – |
| Others (46) | – | – | 4 | – | – | – |
| HCC (18) | 2 | – | – | 1 | 1 | – |
| HCC | – | 5 | – | – | 3 | 1 |
| HCC | – | – | 6 | – | – | 1 |

had HBV-DNA in the liver and one of these showed markers of past infection. Among their eight patients with HCC, one had HBV-DNA in the presence of anti-HBs and anti-HBc, but had been HBsAg positive 4 years previously (Giacchino *et al.*, 1987). Similarly Harrison *et al.* (1986), in a large series involving 160 patients, concluded that there was no evidence to implicate HBV in the pathogenesis of HCC in the absence of HBV seropositivity.

It is possible that the DNA sample from patient C contained dimeric HBV-DNA without an EcoR1 site, in which case the HBV-DNA would have migrated to the same position in each lane; we have noted the same phenomenon previously (Fagan *et al.*, 1986). This would not have been expected if the HBV-DNA were integrated. Certainly the specificity of these bands was confirmed by probing with vector, and the activities of EcoR1 and Hind III by observation of ethidium bromide stained DNA digests.

Other investigators (Hino *et al.*, 1985; Fowler *et al.*, 1986; Pasquinelli *et al.*, 1986) have been unable to detect hepatic HBV-DNA in the absence of serum markers or HBV-RNA transcripts (Yokosuka *et al.*, 1986). A similar situation occurred when using immuno-histochemistry (Blum *et al.*, 1984; Tur-Kaspa *et al.*, 1986) and *in situ* hybridisation in combination with molecular hybridisation (Blum *et al.*, 1983). Liver HBV-DNA sequences have been detected in patients with putative NANB hepatitis (Figus *et al.*, 1984), but we, like Harrison *et al.* (1986), were unable to confirm these findings in any of our nine presumed NANB patients.

We cannot be certain why in six of our HBsAg/HBeAg, with presumed replicating virus, we could detect no hepatic HBV-DNA sequences. However, two patients were positive for $\delta$ virus infection, which has been shown to suppress HBV viral replication, either as a concurrent or as a superimposed infection (Rizetto *et al.*, 1984); one of two patients treated with interferon, which has been reported as a successful antiviral agent (Dusheiko *et al.*, 1986; Alexander *et al.*, 1987), cleared HBeAg from the serum within one year. Two other patients were serum DNA polymerase negative, and thus might have been clearing the virus spontaneously. Another explanation may be the unequal distribution of cellular HBV throughout the liver (Ogata *et al.*, 1988), as well as the possibility of biopsy sampling error. However, if this occurred it was consistent throughout, as the percentages of liver HBV-DNA in the different populations/high risk groups closely parallels the frequency of HBsAg seropositivity.

The intimate epidemiological association between HBV infection and HCC development (Nordenfeldt *et al.*, 1982), and the high incidence of HCC in HBV related cirrhosis (Arthur *et al.*, 1984) have led to suggestions that HBV represents an oncogenic agent in the human liver. Against this, is the observation that non-hepatic tissues and cells which contain the HBV genome, albeit in fewer numbers do not seem prone to malignant transformation (Lie-Injo *et al.*, 1983; Davison *et al.*, 1987). It has been suggested (Gu, 1988) that the integration of HBV-DNA into the host chromosomes corresponds to an initiation event similar to that found in experimental carcinogenesis when using aflatoxin B1 or diethylnitrosamine. This on its own need not cause transformation, but any continuous stimulation of cell proliferation and its subsequent cell division might cause integrated HBV-DNA to become susceptible to a disarrangement of molecular sequences, causing clonal expansion and initiating malignant transformation (Berman, 1988).

From our results, it appears that in order to elucidate the mechanism of HBV and its action at molecular level, emphasis must be placed on the investigation of those subjects in whom serum markers of HBV infection can be demonstrated. It is clear from this and other recent studies that there is currently little evidence to implicate HBV in the pathogenesis of HCC in the absence of serum markers, although this situation may change when more sensitive techniques such as the polymerase chain reaction (PCR) are applied.

We are indebted to the Cancer Research Campaign for their continuing support.

### References

ALEXANDER, G.J.M., BRAHM, J., FAGAN, E.A., DANIELS, H.M., EDDLESTON, A.L.W.F. & WILLIAMS, R. (1987). Loss of HBsAg with interferon therapy in chronic hepatitis B virus infection. *Lancet*, **ii**, 66.

ARTHUR, M.J.P., HALL, A.J. & WRIGHT, R. (1984). Hepatitis B, hepatocellular carcinoma and strategies for prevention. *Lancet*, **i**, 607.

BEASLEY, R.P. (1982). Hepatitis B virus as the aetiologic agent in hepatocellular carcinoma – epidemiologic considerations. *Hepatology*, **2**, S21.

BERMAN, J.J. (1988). Cell proliferation and the aetiology of hepatocellular carcinoma. *J. Hepatol.*, **7**, 305.

BLUM, H.E., HAASE, A.T. & VYAS, G.N. (1984). Molecular pathogenesis of hepatitis B virus infection: simultaneous detection of viral DNA and antigens in paraffin-embedded sections. *Lancet*, **i**, 771.

BLUM, H.E., STOWRING, L., FIGUS, A. *et al.* (1983). Detection of hepatitis B virus DNA in hepatocytes, bile duct epithelium and vascular elements by 'in situ' hybridization. *Proc. Natl Acad. Sci. USA*, **80**, 6685.

BRECHOT, C., NALPAS, B., COUROUCE, A.-M. & 5 others (1982). Evidence that hepatitis B virus has a role in liver-cell carcinoma in alcoholic liver disease. *N. Engl. J. Med.*, **306**, 1384.

BRECHOT, C., DEJEAN, A. & TIOLLAIS, P. (1983). Hepatitis B viral DNA sequences in the infected tissues. *Prog. Clin. Biol. Res.*, **143**, 345.

BRECHOT, C., HADCHOUEL, M., SCOTTO, J. & 4 others (1981). Detection of hepatitis B virus DNA in liver and serum: a direct appraisal of the chronic carrier state. *Lancet*, **ii**, 765.

BRECHOT, C., DEGOS, F., LUGASSY, C. & 10 others (1985). Hepatitis B virus DNA in patients with chronic liver disease and negative tests for hepatitis B surface antigen. *N. Engl. J. Med.*, **312**, 270.

DAVISON, F., ALEXANDER, G.J.M., TROWBRIDGE, R., FAGAN, E.A. & WILLIAMS, R. (1987). Detection of hepatitis B virus in spermatozoa, urine, saliva and leucocytes of chronic HBsAg carriers. *J. Hepatol.*, **4**, 37.

DUSHEIKO, G.M., PATERSON, A.C., PITCHER, L. & 4 others (1986). Recombinant Leucocyte Interferon treatment of chronic hepatitis B. *J. Hepatol.*, **3**, S199.

FAGAN, E.A., ALEXANDER, G.J.M., DAVISON, F.D. & WILLIAMS, R. (1986). Persistence of free HBV DNA in secretions and liver despite loss of serum HBV DNA after interferon induced seroconversion. *J. Med. Virol.*, **20**, 183.

FIGUS, A., BLUM, H.E., VYAS, G.N. & 5 others (1984). Hepatitis B viral nucleotide sequences in non-A, non-B or hepatitis B virus-related chronic liver disease. *Hepatology*, **4**, 364.

FONG, T.-L., GOVINDARAJAN, S., VALINLUCK, B. & REDEKER, A.G. (1988). Status of hepatitis B virus DNA in alcoholic liver disease: a study of a large urban population in the United States. *Hepatology*, **8**, 1602.

FOWLER, M.J.F., GREENFIELD, C., CHU, C.-M. & 8 others (1986). Integration of HBV-DNA may not be a prerequisite for the maintenance of the state of malignant transformation. *J. Hepatol.*, **2**, 218.

GIACCHINO, R., PONTISSO, P., NAVONE, C. & 4 others (1987). Hepatitis B virus (HBV)-DNA positive hepatocellular carcinoma following hepatitis B virus infection in a child. *J. Med. Virol.*, **23**, 151.

GU, J.-R. (1988). Molecular aspects of human hepatic carcinogenesis. *Carcinogenesis*, **9**, 697.

HARRISON, T.J., ANDERSON, M.G., MURRAY-LYON, I.M. & ZUCKERMAN, A.J. (1986). Hepatitis B virus DNA in the hepatocyte – a series of 160 biopsies. *J. Hepatol.*, **2**, 1.

HINO, O., KITAGAWA, T. & SUGANO, H. (1985). Relationship between serum and histochemical markers for hepatitis B virus and rate of viral integration in hepatocellular carcinomas in Japan. *Int. J. Cancer*, **35**, 5.

LASKY, R.A. & MILLS, A.D. (1977). Enhanced autoradiographic detection of $^{32}$P and $^{125}$I, using intensifying screens and hypersensitized film. *FEBS Lett.*, **82**, 314.

LIE INJO, L.E., BALASEGARAM, M., LOPEZ, C.G. & HERRERA, A.R. (1983). Hepatitis B virus DNA in liver and white cells of patients with hepatoma. *DNA*, **22**, 301.

MANIATIS, T., FRITSCH, G.F. & SAMBROOK, J. (1982). *Molecular Cloning. A Laboratory Manual*. Cold Spring Harbor Laboratory: New York.

NALPAS, B., BERTHELOT, P., THIERS, B. & 4 others (1985). Hepatitis B virus multiplication in the absence of usual serological markers – a study of 146 chronic alcoholics. *J. Hepatol.*, **1**, 89.

NORDENFELDT, E., LINDHOLM, T., LOEFGREN, B. *et al.* (1982). Different categories of chronic HBsAg carriers: a long term follow up. In *Viral Hepatitis*, Szmuness, W., Alter, H.J. & Maynard, J.E. (eds) p. 237. Franklin Institute Press: Philadelphia.

OGATA, N., KOJIMA, T., OKOSHI, S., KAMIMURA, T., ICHIDA, F. & HAMADA, C. (1988). Mode of integration of hepatitis B virus DNA in chronically infected livers with indications of multiclonal growth of hepatocytes and some hepatoma cells. In *Viral and Liver Diseases*, Zuckerman, A.J. (ed.) p. 746. Alan R. Liss: New York.

PASQUINELLI, C., LAURE, F., CHATENOUD, L & 7 others (1986). Hepatitis B virus DNA in mononuclear blood cells. *J. Hepatol.*, **3**, 95.

PONTISSO, P., STENICO, D., DIODATI, G. & 5 others (1987). HBV-DNA sequences are rarely detected in the liver of patients with HBsAg-negative chronic active liver disease and with hepatocellular carcinoma in Italy. *Liver*, **7**, 211.

POPPER, H., SHAFRITZ, A. & HOOFNAGLE, J.H. (1987). Relation of Hepatitis B carrier state to hepatocellular carcinoma. *Hepatology*, **7**, 764.

RIGBY, P.W.J., DIECKMANN, M., RHODES, C. & BERG, P. (1977). Labelling deoxyribonucleic acid to high specific activity 'in vitro' by nick translation with DNA polymerase I. *J. Mol. Biol.*, **113**, 237.

RIZETTO, M., HAYER, B.H., PURCELL, R.H. *et al.* (1984). Hepatitis virus infection. In *Viral Hepatitis and Liver Disease*, Vyas, G.N. (ed.) p. 371. Grune & Stratton: Orlando, FL.

SHAFRITZ, D., SHOUVAL, D., SHERMAN, H.I., HADZIYANNIS, S.J. & KEW, M.C. (1981). Integration of hepatitis B virus DNA into the genome of liver cells in chronic liver disease and hepatocellular carcinoma: studies in percutaneous liver biopsies and post-mortem tissue specimens. *N. Engl. J. Med.*, **305**, 1067.

SOUTHERN, E.M. (1975). Detection of specific sequences among DNA fragments separated by electrophoresis. *J. Mol. Biol.*, **98**, 503.

SZMUNESS, W., HARLEY, E.J., IKRAM, H. & STEVENS, C.E. (1978). Sociodemographic aspects of the epidemiology of hepatitis B. In *Viral Hepatitis*, Vyas, G.N., Cohen, S.N. & Schmid, R. (eds) p. 297. Franklin Institute Press: Philadelphia.

TUR-KASPA, R., BURK, R.D., LIEBERMAN, H.H. & SHAFRITZ, D.A. (1986). Hepatitis B virus gene expression in relation to virus replication and HBV-DNA integration. *J. Hepatol.*, **3**, S25.

VERGANI, D., LOCASCIULLI, A., MASERA, G. & 6 others (1982). Histological evidence of hepatitis B virus infection with negative serology in children with acute leukaemia who develop chronic liver disease. *Lancet*, **i**, 361.

WAHL, G.M., STERN, M., STARK, G.R. *et al.* (1979). Efficient transfer of large DNA fragments from agarose gels to diazobenzyloxy methyl paper and rapid hybridisation by using dextran sulphate. *Proc. Natl Acad. Sci. USA*, **76**, 3683.

WALTER, E., BLUM, H.E., MEIER, P. & 6 others (1988). Hepatocellular carcinoma in alcoholic liver disease: no evidence for a pathogenetic role of hepatitis B virus infection. *Hepatology*, **8**, 745.

YOKOSUKA, O., OMATA, M., IMAZEKI, F., ITO, Y. & OKUDA, K. (1986). Hepatitis B virus RNA transcripts and DNA in chronic liver disease. *N. Engl. J. Med.*, **315**, 1187.